# Modified mRNA as a Therapeutic Tool for the Heart

**DOI:** 10.1007/s10557-020-07051-4

**Published:** 2020-08-21

**Authors:** Keerat Kaur, Lior Zangi

**Affiliations:** 1 Cardiovascular Research Center, Icahn School of Medicine at Mount Sinai, New York, NY USA; 2 Department of Genetics and Genomic Sciences, Icahn School of Medicine at Mount Sinai, New York, NY USA; 3 Black Family Stem Cell Institute, Icahn School of Medicine at Mount Sinai, New York, NY USA

**Keywords:** Modified mRNA, Gene therapy, Myocardial infarction, Cardiovascular regeneration, Cardiac protection, Cardiomyocyte proliferation

## Abstract

Despite various clinical modalities available for patients, heart disease remains among the leading causes of mortality and morbidity worldwide. Genetic medicine, particularly mRNA, has broad potential as a therapeutic. More specifically, mRNA-based protein delivery has been used in the fields of cancer and vaccination, but recent changes to the structural composition of mRNA have led the scientific community to swiftly embrace it as a new drug to deliver missing genes to injured myocardium and many other organs. Modified mRNA (modRNA)–based gene delivery features transient but potent protein translation and low immunogenicity, with minimal risk of insertional mutagenesis. In this review, we compared and listed the advantages of modRNA over traditional vectors for cardiac therapy, with particular focus on using modRNA therapy in cardiac repair. We present a comprehensive overview of modRNA’s role in cardiomyocyte (CM) proliferation, cardiac vascularization, and prevention of cardiac apoptosis. We also emphasize recent advances in modRNA delivery strategies and discuss the challenges for its clinical translation.

## Genetic Medicine in Heart Disease

Completed in 2003, the overwhelming success of the Human Genome Project enabled researchers across the globe to identify and sequence all genes present in human DNA. The ability to analyze target genes and related signaling pathways made gene therapy a novel form of molecular medicine. Conceptually, gene therapy is quite straightforward: introducing a normal gene into a cell involved in a disease process should enable that gene’s protein product to correct or slow the disorder’s advancement. Based on this idea, gene therapy aims to deliver genetic material to manage both inherited and acquired diseases.

Although pharmacological therapeutic approaches developed over the past 50 years have considerably improved quality of life for patients with heart disease, these common pharmacological interventions (β-blockers, angiotensin-converting enzyme inhibitors, angiotensin receptor II-antagonists, and diuretics) do not interact with or demonstrably curtail the relevant underlying intracellular signal transduction mechanisms that cause or intensify the development and progression of heart disease [1, 2]. To date, there is no known cure for heart failure (HF), and the limited numbers of hearts available for transplantation are not a sufficient solution. As a result, the morbidity and mortality rates of heart disease remain unacceptably high in Western industrialized countries and are rising in others [3]. Thus, to fill this gap in modern pharmacologic therapies and better assist patients with heart failure patients, novel remedies are badly needed.

Gene therapy presents an ideal approach for manipulating molecular mechanisms that cannot be altered pharmacologically. Also, gene therapy offers spatially and temporally effective gene delivery to the myocardium, an approach that can provide lasting benefits in organ-wide chronic and progressive HF. In the case of ischemic heart disease (IHD), gene delivery ideally should (a) induce cardiac regeneration via cardiomyocyte proliferation, (b) prevent cardiac cell death (CMs or non-CMs), (c) target endothelial cells (ECs) to stabilize coronary plaques and induce angiogenesis, (d) trigger cardiac reprogramming through fibroblasts, and (e) reduce electrophysiological abnormalities. Gene delivery vehicles should have an appropriate packaging system to convey the genetic material into a variety of cells, with high efficiency and for a desirable period of time.

## Current Approaches to Achieve Gene Therapy Goals

Current gene delivery vehicles fall into two broad categories: viral and non-viral gene delivery vectors. Conventional viral vectors used in cardiovascular diseases, including retroviruses, lentiviruses, and adenovirus-associated virus (AAV), can efficiently deliver genes into cardiac cells; however, every known viral vector bears some risks and limitations. By contrast, non-viral vectors, which are comprised of 2 categories: physical (plasmid DNA, electroporation, sonoporation, hydrodynamic, ultrasound, magnetofection, gene gun) and chemical (cationic lipids, different cationic polymers, Chitosan, dendrimers, lipid polymers, inorganic nanoparticles, cell-penetrating peptides), are safer to use but have limited transfection efficiency and require proper structure with specific absorption efficiency to provide effective means of gene delivery [4, 5]. In this regards, novel mRNA-based therapy is a promising gene delivery platform for cardiac gene therapy.

I. *Gene packaging and delivery:* The expression level of the gene in the target cells is controlled by regulatory elements (promoters and enhancers) packaged alongside the gene inside the viral protein coat. Thus, the space available in the capsid determines the size of the therapeutic gene to be delivered. Adenoviruses (AV) and lentiviruses have a relatively large insert capacity and contain a genome of approximately 36 kb and 14 kb, respectively, while smaller viruses, like AAV, have a 25-nm diameter protein coat and a much smaller insert capacity space (only ~ 5 kb), which limits the size of the therapeutic gene [6–8]. In this context, non-viral vectors like naked DNA plasmid or modRNA do not have any size constraint and can be useful in carrying and delivering a therapeutic gene of any size directly to cardiac cells. Given the fact that gene expression reduces in correlation with an increase in the size of mRNA, modRNA provides the flexibility for controlling the amount of gene delivery in the cells. Furthermore, modRNA delivery is not influenced by the state of the nuclear membrane and can thus transfect both dividing and non-dividing cells, a trait most viral vectors lack.
II. *Gene expression pharmocokinetics:* The temporal expression patterns of therapeutic genes are critical to whether the gene transfer system can be employed for efficient and positive recovery. Because every disorder requires unique temporal expression, it is desirable to choose an optimal vector that can deliver genes within a particular time frame for appropriate protein turnover. Viral vectors like lentiviruses provide strong gene expression for an extended period of time and are popular choices for treating pathophysiologies that need lifelong expression of a missing protein. In a heart failure model, prolonged expression of sarcoplasmic reticulum Ca^2+^ ATPase via pump with lentivirus injection was reported to improve myocardial function in mice [9]. Over the last decade, various pre-clinical studies have explored using AAV in prolonged replacement of genes involved in inherited heart disorders. AAV-assisted Sumo-1 gene transfer into pig hearts was shown to improve their cardiac function post-injury [10], as AAV-assisted gene expression peaks after 4 weeks and continues up to 11 months [11]. However, uncontrolled and prolonged gene delivery can pose unnecessary risks when only transient expression of an appropriate gene is needed to trigger an underlying signaling pathway. Further, as significant changes occur in cardiac cells as early as 24 h post-infarction, early and quick interventions are needed to prevent and protect the heart from further damage. Accordingly, modRNA’s unique pulse-like, immediate gene expression is highly favorable in preventing cardiac remodeling post-MI. ModRNA gene therapy has now been shown to prevent cardiomyocyte death [12, 13] and induce cardiomyocyte and vascular proliferation without risking uncontrolled cell division or tumor formation. In 2013, Zangi et al. successfully showed vascular regeneration after MI with modRNA-induced VEGFA expression [14].
III. *Gene transfer efficiency:* Efficient gene transfer into the cell is vital to successful gene translation and depends on properties of the vector used for transfection. Viral vectors depend on vector infectivity, promotor control over the gene of interest, the viral vector’s affinity to membrane receptors, receptor availability, and foreign gene inactivation by the host cell. In the failing heart, endogenous molecular mechanisms in cardiac cells change, which may result in the delivered gene being silenced despite its active form, thereby substantially reducing therapeutic gene expression. Effective gene therapy thus requires a viral vector with high infection multiplicity that can transfer a high number of viral particles to the targeted cardiac cell in order to achieve the desired functional effect. mRNA-based therapies are proven to be successful in this difficult context, as therapeutic gene amounts can be finely controlled. Moreover, this technology can deliver gene combinations with ratios tailored to the targeted cell. In the case of gene delivery assisted by viral vectors, the target gene must be translocated to the cell nucleus, where it then interacts with the array of nuclear proteins that regulate gene expression. Using mRNA transfection overcomes the need for nuclear localization to induce transcription, enabling mRNA therapy to efficiently translate the desired gene without other influencers.
IV. *Potential safety concerns*: Gene delivery system safety must be thoroughly determined before vehicles can be selected for myocardial gene therapy. Using viruses for gene therapy raises a number of safety concerns. AVs can trigger a strong innate immune response and toxicity due to viral gene products. The use of AVs came into serious question in 1999 after a patient with ornithine transcarbamylase deficiency died due to a massive immune response following the injection of an AV vector [15]. Lentivirus vectors are of limited use in cardiovascular disorders because these viruses randomly integrate into the host with a preference for targeting coding regions, thus creating a huge risk of insertional mutagenesis and oncogenesis [16]. While AAVs are highly favored over other vectors due to their lack of immunogenicity, a critical obstacle in AAV gene therapy translation is the presence of preexisting neutralizing anti-AAV antibodies that are present in 30 to 50% of the population [17]. In addition to ease of vector production and reduced limitations on expression cassette size, non-viral gene delivery is also a safer option under several physiological conditions. modRNA non-viral gene delivery shows minimal biosafety risks, as the mRNA does not integrate into the host genome. Moreover, mRNA offers transient gene expression, which minimizes the risk of mutagenesis after mRNA therapy. A side-by-side comparison of commonly used vectors for cardiac repair can be found in Table 1.

**Table 1:** Various gene delivery vectors for cardiac repair

Delivery method	Vehicle diameter	Gene packaging capacity	Expression kinetics	Immunogenicity	Major drawback
ModRNA	Variable	Unlimited	Short term expression up to 2–7 days	Minimal	Transient expression
Plasmid	Variable	Unlimited	Expression up to 2 months	Minimal	Low transfection efficiency
Lentivirus	90 nm	~ 8 kb	Long-term cardiac expression	Moderate	Risk of insertional mutagenesis
AAV	25 nm	~ 5 kb	Long-term cardiac expression up to 11 months	Mild	Risk of neutralizing antibodies and T cell responses
Adenovirus	100 nm	~ 36 kb	Expression up to 2 weeks	Strong	High antibody and inflammatory response

## The Popularity of Messenger RNA

mRNA, a naturally occurring molecule, holds revolutionary medical potential as it can efficiently and accurately translate the information from DNA into proteins, thus allowing the host to generate their own personalized medicine. The concept of mRNA-based gene transfer in mammalian cells in vitro was first introduced by Bhargava and Shanmugam in 1970 [18], and almost two decades later, mRNA encoding for reporter gene B galactosidase was successfully injected intramuscularly in a mouse model [19]. Although its capacity to self-amplify made mRNA therapy useful in vaccine development [20], it was not considered a therapeutic entity and was believed to be highly therapeutic unsuitable due to the immune response it elicited [21]. Upon entry into the cell, in opposed from coming out from the nucleus, unmodified mRNAs are recognized by the innate immune system via recognition receptors, known as toll-like receptors (TLRs) 7 and 8, located in the endosome [22]. Activation of TLRs leads to the production of pro-inflammatory cytokines and type I interferons that activate the endonuclease (RNaseL), which ultimately degrades the imported mRNA [23, 24], shutting downs its translation. This process meant synthetic mRNA had limitations in gain-of-function studies.

**Fig. 1:**
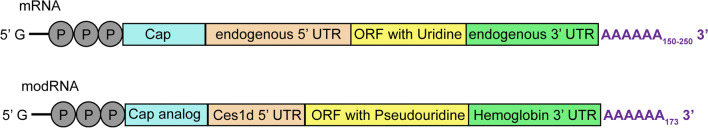
Schematic illustration showing the modifications made to mRNA’s structure to increase its translation and stability

Yet modifying mRNA’s secondary structure (Fig. 1), particularly changing uridine with naturally occurring pseudouridine and 5-methyl-cytosine for cytosine [25], combined with advances in in vitro transcription technologies led to less recognition by TLRs and nucleases. Additionally, the stability and in vivo translation efficiency of modRNA have been increased by capping the molecule with the 3′-O-Mem7G(5′)ppp(5′)G Anti Reverse Cap Analog (ARCA) at its 5′end [26, 27] and replacing 5′UTR with one from the fatty acid metabolism gene carboxylesterase 1D (Ces1d) [28]. ModRNA has the capacity to target the gene of interest via multiple mechanisms including: (i) gain of function by overexpressing a target molecule [13, 29], (ii) loss of function by either using dominant negative molecules or introducing miRNA [30], (iii) correcting gene deletions at the mRNA level [31], and (iv) inhibiting intracellular signaling pathways by introducing decoy receptors to dilute the ligands [30].

## Modified mRNA Therapy in the Heart

During MI, the occlusion of the coronary artery leads to ischemia and subsequent loss of CMs and ECs, which are quickly replaced by highly proliferating fibroblasts, resulting in scarring and remodeling of heart tissue in the affected areas. Due to their limited proliferation capacity, surviving CMs are incapable of reversing the damage and replace the dead CMs. Moreover, the damage in the coronary vasculature creates an unfavorable milieu for CM survival. These irreversible changes in the heart along with increased oxidative stress and inflammation lead to impaired pump function and, ultimately, heart failure. ModRNA is a promising gene therapy approach that can therapeutically target several mechanisms that may protect MI survivors against HF. Studies have proven three major strategies by which modRNA can be used to treat ischemic injury: (i) inducing CM proliferation, (ii) inhibiting heart cell death and attenuating inflammation, and (iii) supporting cardiovascular regeneration. The uses of modRNA technology as a therapeutic approach for cardiac repair are listed in Table 2.

I. *CM proliferation by modRNA approach*: When delivered at the time of MI, modRNA is an effective vehicle for re-awakening CM proliferation. In 2018, Magadum et al. published the first report noting modRNA can induce CM proliferation and regeneration by upregulating mutated human follistatin-like (hFSTL1). This work shows that mutation at the N-glycosylation site, position 180 of asparagine (N) with glutamine (Q), was sufficient and necessary to activate CM proliferation and reduce cardiac remodeling post-MI. Post-translational modification, i.e., glycosylation of hFSTL1 upon N180 site ablation, by allowing it to activate unknown receptors in the heart was hypothesized to be responsible for CM regeneration in vitro or neonatal rats or adult mice after MI with no indications of cardiac hypertrophy. Further, a single dose of N180Q hFSTL1 modRNA to the mouse myocardium post-MI significantly improved cardiac function, decreased scar size, and increased capillary density after 28 days, showing the effectiveness of modRNA in triggering CM proliferation and cardiac regeneration [12].

**Table 2:** Key studies identifying modRNA as cardiac repair therapy

Focus area	Publication	Protein target	Experimental outcome	Delivery vehicle	Administration method	Reference
Inducing CM proliferation	Magadum et al.	mutated FSTL1	CM proliferation, decreased scar size, improved heart function	Sucrose-citrate buffer	Intracardiac injection	[12]
	Magadum et al.	Pkm2	Induced CM cell cycle, reduced oxidative stress	Sucrose-citrate buffer	Intracardiac injection	[13]
Inhibiting cardiac apoptosis/enhancing survival	Huang et al.	IGF-1	Reduced cell apoptosis/promoted cell survival	Polyethylenimine-based nanoparticle	Intracardiac injection	[32]
	Zangi et al.	DN-IGF-1R, IGFR	Reduced cell differentiation into adipocytes post-MI	RNAiMAX	Intracardiac injection/gel application	[30]
	Hadas et al.	AC	Increased cell survival, improved cardiac function and mice survival	Sucrose-citrate buffer	Intracardiac injection	[29]
	Chen et al.	aYAP	Decreased CM necrosis, attenuated innate immune responses	Saline	Intracardiac injection	[33]
Inducing cardiovascular regeneration	Zangi et al.	VEGFA	Induced angiogenesis, improved myocardial function and mice survival	RNAiMAX	Intracardiac injection	[14]
	Lui et al.	VEGFA	Endothelial specification engraftment, proliferation, and reduced apoptosis of the human Isl1+ progenitors in vivo	RNAiMAX	Matrigel, subcutaneous injection	[34]
	Carlsson et al.	VEGFA	Increased capillary density, decreased fibrosis and improved heart function post-MI	Sucrose-citrate buffer	Intracardiac injection	[35]
	Moderna Therapeutics	VEGFA	Not reported	Citrate buffer saline	Epicardial injection	[36]
ModRNA delivery and production optimization	Turnbull et al.	EGFP	Efficient modRNA delivery to the heart	Formulated lipidoid nanoparticles (FLNP)	Intramyocardial/intracoronary injection	[37]
	Turnbull et al.	EGFP	Protocol	Formulated lipidoid nanoparticles (FLNP)		[38]
	Kondrat et al.	varies	Protocol	RNAiMAX		[39]
	Sultana et al.	Luciferase	Optimized modRNA amount, time and delivery	Sucrose-citrate buffer	Intracardiac injection	[40]
	Singh et al.	EGFP, mCherry, Fluc	Optimized modRNA delivery into myocardium	Alginate, nanomaterial encapsulated	Intracardiac injection	[41]
	Hadas et al.	GFP, Luciferase	Improved modRNA yield and translation efficiency, reduced its immunogenicity	Sucrose-citrate buffer	Intracardiac injection	[27]
	Sultana et al.	Luciferase	Increased translation by replacing 5’UTR	Sucrose-citrate buffer	Intracardiac injection	[28]

In a subsequent study, Zangi and colleagues showed that modRNA technology can induce the CM cell cycle by upregulating the glycolytic enzyme Pkm2 (pyruvate kinase muscle isoenzyme 2) [13]. Pkm2 is primarily expressed at higher levels in regenerative fetal heart and neonatal CMs but not in adult CMs. This work established that Pkm2 can regulate cell cycle progression by elevating anabolic metabolism in CMs (via pentose phosphate pathway) interacting with β-catenin and upregulating its downstream targets Cyclin, D1, and C-Myc. Further, they demonstrated that Pkm2 plays a role in regulating oxidative stress by reducing ROS production post-MI. ModRNA-mediated Pkm2 elevation re-invigorated the CM cell cycle, which led to CM cell division and subsequent cardiac regeneration. Using the lineage-tracing mouse model and relabeling the CMs, the study showed that the ectopic expression of modRNA encoding the Pkm2 gene increased cardiomyocyte cell division in adult mice and suppressed postnatal CM cell cycle arrest. Moreover, Pkm2 modRNA delivery led to significantly improved cardiac function and outcomes after acute or chronic myocardial infarction, thus rescuing cardiac remodeling.

II. *ModRNA offers cardiac protection*: After ischemic stress in the mammalian heart tissue, progressive death of heart cells in the left ventricle results in deteriorated cardiac function. A powerful predictor of heart failure in IHD patients is high concentrations of simple membrane sphingolipids, known as ceramides, in the plasma. More specifically, elevated ceramide levels in the blood are associated with programmed cell death and higher probability of MI recurrence. Considering the role of ceramides and the enzyme associated with their metabolism in heart disease progression, they may also affect CM death. As was recently reported, modRNA-delivered acid ceramidase (AC) overexpression is associated with lower CM death rates and increased cell survival after hypoxia or MI. AC primarily frees fatty acids and sphingosine in ceramide hydrolysis, and elevated AC has been reported to lessen the negative effects of elevated ceramides. In addition, elevated levels of this enzyme reduced detrimental neutrophil levels in the LV, thus decreasing the inflammation associated with MI and promoting cell survival. Indeed, AC-modRNA–treated mice showed significantly better heart function, smaller LV scars, and longer survival in post-ischemic injury [29].

ModRNA gene delivery has also been used to ease IR-induced inflammation. As the first response to cardiac injury, innate immune system activation recruits leukocytes, including damage-associated molecular patterns, cytokines, and chemokines, that help coordinate dead and damaged cell removal, clear extracellular matrix debris, revascularize, and form scars. On the downside, these recruited leukocytes also activate the downstream signaling pathways, resulting in cell necrosis [42]. Chen et al. showed that modRNA-mediated transient activation of transcriptional co-activator yes-associated protein (aYAP), already known to promote cell proliferation and survival, attenuated the inflammatory innate immune response by lowering neutrophil infiltration after IR injury. This efficient expression of aYAP protein via modRNA treatment led to improved heart function, reduced CM necrosis, diminished scar size, and prevented hypertrophic cardiac remodeling [33].

Huang and group showed the role of modRNA-delivered insulin-like growth factor-1 (IGF1) in providing cardioprotection in CM after ischemia and MI [32]. Using modRNA to deliver IGF1 to the at-risk area in mouse hearts post-MI promoted CM survival and limited cell death under hypoxia-induced apoptosis conditions. A polyethylenimine-based nanoparticle-assisted modRNA delivery of IGF-1 showed rapid protein expression as early as 2 h after injection and peaked after 24 h. Elevated IGF-1 protein levels led to phosphorylation of downstream targets Akt and Erk, decreasing cell apoptosis by 50%, as seen by lower levels of TUNEL-positive cells and reduced caspase-9 activity in the mouse model following ischemic injury and hypoxia. By contrast, IGF-1 upregulation triggers epicardial progenitor cells to differentiate into adipogenic cells, leading to epicardial adipose tissue (EAT) formation post-MI. Further downregulating the IGF-1 signaling pathway by delivering dominant-negative IGF-1 receptor antagonists reversed EAT formation in the heart [30]. Overall, these studies demonstrate that transient protein expression driven by modRNA gene delivery has the potential to enact an extended cytoprotective effect.

III. *ModRNA therapy induces cardiovascular regeneration*: Post-MI, tissue ischemia develops around the infarction site due to loss of ECs and subsequent reduction of vascularization. One established way to repair this damage is to regenerate the blood vessels supporting the CMs. Various studies have shown the potency of VEGFA as an angiogenic factor after ischemic injury. However, the clinical trials addressing VEFGA delivered via adenoviral plasmid or recombinant protein as a cardiac therapy showed only moderate improvements in cardiac function. One explanation for this lack of vascular regeneration is the inefficient delivery platform, which led to either protein degradation by proteases or off-target delivery due to systemic injections [43]. Another issue with prolonged VEGFA expression is its negative effects on vascular permeability, as increased VEGFA is associated with development of leaky, immature vessels with elevated vessel permeability, and pool perfusion rate [44].

To overcome the problems related to sustained protein delivery with viral vectors, Zangi et al. [14] explored efficient pulse-like delivery of VEGFA in a mouse MI model. This study demonstrated that modRNA-induced VEGFA expression increased the levels of endogenous heart progenitors, mobilized their migration into the myocardium, and redirected their differentiation toward cardiovascular lineages. Further, transient VEGFA expression derived from modRNA was superior to plasmid DNA in reducing infarct size, enhancing myocardial perfusion and improving survival. These findings verified that modRNA therapy might be well suited to delivering paracrine factors that enhance cardiac regeneration.

**Fig. 2:**
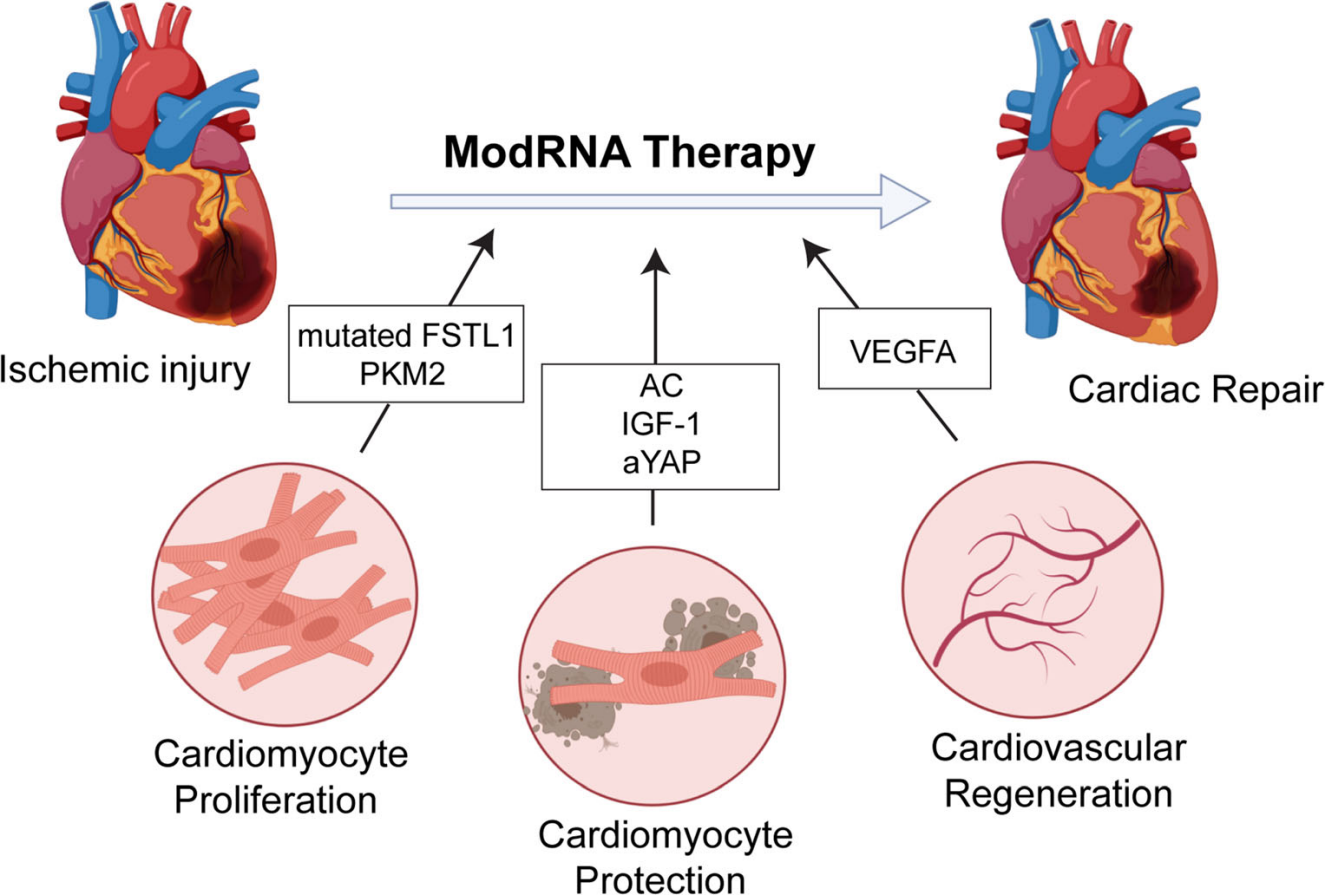
Direct cardiac repair using modRNA system to deliver genes involved in inducing cardiomyocyte proliferation, cardiomyocyte protection, and cardiovascular regeneration

Wide screening of angiocrine factors expressed in ECs derived from the outflow tract of human fetal hearts compared with ECs derived from human cord blood revealed that VEGFA is the key factor involved in differentiating human ESC-derived Isl1+ progenitors toward an EC fate. Lui et al. reported that modRNA-driven pulse-like VEGF-A overexpression not only caused endothelial specification but also engraftment, proliferation, and survival (reduced apoptosis) of the human Isl1+ progenitors in vivo [34]. Promising results from a study conducted in swine moved the field one step closer to taking modRNA-delivered VEGFA intervention to the clinic [35]. Intracardiac delivery of VEGFA mRNA improved left ventricular ejection fraction, inotropy, and ventricular compliance, with increased border zone arteriolar and capillary density 2 months after permanent occlusion surgery. This modRNA-based single-dose VEGFA delivery proved sufficient to improve ventricular function and curtail myocardial damage in mini pigs. In addition to these promising results, Moderna, an mRNA-based company, and its partner AstraZeneca are examining the effect of modRNA-delivered VEGFA delivery in patients with moderately impaired systolic function undergoing coronary artery bypass grafting surgery (clinical trial number NCT03370887) [36]. This phase II clinical trial is investigating the safety and tolerability of VEGFA modRNA epicardial injection in patients. Once positive results are received, modRNA-delivered VEGFA therapy may come to represent a new class of therapies for improving cardiac function after ischemic injury. Figure 2 illustrates the mechanisms by which modRNA offers cardioprotection to the injured myocardium.

## ModRNA Delivery and Translation Systems

Multiple methods and vehicles have been used for intracellular modRNA delivery to cells in vitro and in vivo. Traditionally, physical transfection methods like electroporation and gene gun or microinjections have been shown to efficiently deliver modRNA to cells; however, these methods were limited to cancer therapy and, to a lesser extent, protein replacement therapy due to their high costs and invasive nature [45]. As an alternate option, cationic lipid or polymers, which make an electrostatic bond with the negatively charged nucleic acid, have shown considerable promise for modRNA delivery. Other possible modRNA delivery methods are nanoparticles, which ensure nucleic acid enters the cell membrane by their spherical shape containing polar head groups and non-polar tails. These chemical methods feature high versatility, reduced costs, simpler use, and low toxicity. The common formulations contain four components: an amine-containing lipid or lipid-like material, a phospholipid, cholesterol, and lipid-anchored polyethylene glycol. These compounds are flexible, as the amount of each component can be adjusted to enhance targeted delivery and protection of the conjugated nucleic acid from nuclease degradation. Nanoparticles’ reliability, efficacy, and flexibility make them attractive for modRNA delivery.

Cationic lipoplex formulations, e.g., lipofectamine, have proven to successfully deliver modRNA to isolated CMs in vitro. Transfecting modRNA using positively charged lipofectamine resulted in very high transfection levels in neonatal rat (98.3%) and human pluripotent stem cell-derived CMs (98.9%). Despite such convincing mRNA delivery results in vitro, RNAimax use was limited for in vivo studies due to high cell death rates around intracardiac injection sites [40].

In comparing different modRNA delivery modes, Sultana et al. [40] reported that naked modRNA (with sucrose-citrate buffer) was superior in modRNA translation to lipid nanoparticles. This was associated with lower heart cell death associated. This lead us to believe that naked modRNA is an optimal approach for cardiac gene delivery as the buffer solution may provide viscosity and act as a chelating agent for mRNA preservation [46]. With this strategy, delivering 25–100 μg of modRNA was able to widely translate the gene of interest, covering more than 20% of the myocardium. The rapid expression element of modRNA resulted in protein translation within 10 min of transfection. Carlsson et al. later verified these findings in large animal studies. VEGFA delivery into pig hearts with biocompatible citrate-saline buffer showed tissue-specific protein expression without stimulating any immune response [35]. Hence, delivering free or loosely bound mRNA in cytoplasm leads to higher translation efficiency and opens the possibility of mRNA therapy in acute cardiac diseases.

Another modRNA delivery option was explained by Turnbull et al., who showed successful in vivo modRNA delivery to the myocardium using formulated lipidoid nanoparticles (FLNP) [37, 38]. Intracardiac injection of FLNP carrying modRNA showed reporter gene protein expression peaked 20 h after injection with minimal off-target expression. The authors show modRNA has intracardiac stability, with gene expression detected up to 14 days post-delivery. Further, the group also successfully observed fluorescent signal as early as 20 min after FLNP/eGFPmodRNA injection in the pig heart, confirming the rapid and efficient nature of modRNA-assisted gene delivery. Novel microencapsulation technology has also been tested for modRNA delivery into myocardium of small and large animals [41]. The study reported that using ∼ 100 nm microencapsulated modRNA (M3RNA) lead to protein detection as early as 2–4 h with stability for up to 7 days in isolated CMs and 72 h in murine hearts. M3RNA in a porcine model further indicated rapid, targeted protein expression, thus providing an alternate approach to deliver modRNA into the injured heart.

Although modRNA therapy is now undergoing clinical trials, and numerous pre-clinical trials are also underway, researchers are still finding ways to improve its translational capacity in vivo and develop cost-effective protocols for modRNA production. The 5′ 7-methylguanylate cap plays a role in stabilizing mRNA by protecting it from exonucleases and promoting the translation initiation by binding eukaryotic initiation factor 4E [47]. The addition of Anti reverse cap analogue increases the translation efficiency by ensuring capping occurs in the correct orientation [48]. However, this chemical capping strategy requires a high ratio of ARCA and GTP concentrations to produce a high percentage of capped mRNA. Hadas and colleagues provided an amendment to the ratios of 5′ ARCA cap and previously described N1-methyl-pseudouridine [39], which can cut modRNA production costs and enhance its protein expression. They also optimized the nucleotide concentration to 31.6 mM per reaction, resulting in a 290% higher modRNA yield. This nucleotide optimization led to increased percentage of the capped modRNA, thus reducing its immunogenicity in both human cell lines and primary cardiac cells [27].

In an attempt to further enhance the translational efficiency of modRNA, modifications of other mRNA structural domains provide additional avenues for investigation. Eukaryotic gene expression is regulated by 5′ and 3′UTRs of modRNA, which stabilize mRNA [49] by integrating with the translational machinery or serving as the binding site for micro RNA and mRNA decay-promoting proteins, respectively [50]. In an attempt to increase the half-life of IVT mRNA and its translation into protein, various studies have selected 3′UTRs of α- and β-globin mRNAs and incorporated them into the 3′UTR of IVT mRNA [51]. A recent study showed that replacing traditionally used artificial 5′UTR with one from Ces1d doubled the translation of a modRNA-delivered reporter gene in the heart post-MI. Mechanistically, Ces1d is involved in lipid metabolism, and changes in this metabolic activity under MI conditions trigger Ces1d mRNA, leading to better translation. Further, this altered modRNA structure also enhanced translation in other organs, including the liver, under ischemic conditions [28]. These promising studies thus indicate modRNA technology can be used in future cardiac regenerative applications.

To date, cardiac gene therapy via modRNA has shown the ability to repair injured hearts in various aspects including expressing angiogenic factors for vessel regeneration and sphingolipid metabolic genes to limit and prevent cardiac damage or to induce cell cycle-promoting genes. However, while upregulating these genes can prove to be favorable for one cell type, their overexpression in neighboring cells can produce unfavorable outcomes. For instance, after cardiac injury, there is an urgent need for CMs to undergo cell cycle. The global delivery of cell cycle-promoting genes can also stimulate non-CMs in the heart to undergo division, leading to increased scar formation or eliciting an undesirable immune response. Thus, to overcome these detrimental effects of non-specific delivery of modRNA into cardiac cells, Magadum et al. demonstrated a novel mechanism of desirable gene translation exclusively in CMs upon intracardiac injection. They create a unique circuit modRNA based on archaeal ribosomal protein L7Ae, which suppresses translation of the gene containing a kink-turn motif, a specific binding site for L7Ae. Upon simultaneous transfections of L7Ae and a gene containing kink-turn motif, L7Ae attaches to the binding site and suppresses translation of the gene of interest. Using this cell specific system, the study ensured CM specificity by adding a CM-specific microRNA recognition element to the 3′UTR of the L7Ae gene. This prevented L7Ae translation in CMs that extensively and particularly express those microRNAs, so that the gene of interest translated exclusively in CMs [13]. Thus, this novel, first of its kind, in vivo modRNA model promotes gene of interest homing to the desired cell in the infarcted myocardium.

## Challenges and Future Directions of Cardiac modRNA Therapy

Although modRNA applications in cardiology are progressing quickly, hurdles remain that must be overcome to achieve translation. Various publications have shown that modRNA therapy improves outcomes after MI [52]; however, taking this research to the clinic has been hampered by poorly defined delivery systems. Currently, intracardiac injection is the most effective delivery method for delivering genes to the heart, but this direct gene penetration into the myocardium causes stress and local injury to the tissue. Thus, there is a real need to develop efficient mRNA delivery systems that can ensure targeted, non-invasive gene relay into the heart. There is growing interest in cell-penetrating peptides (CPP) that can be used in conjunction with modRNA to ensure its target-specific delivery. Recent studies have supported the use of CPP to deliver small interfering RNA to inhibit target gene expression in cancer cells [53]. Additionally, mRNA transfection may be efficiently mediated by RNA aptamers, which bind to specific cell markers and can be modified to target specific tissues [54]. Furthermore, it is crucial to optimize consistent dosing across the myocardium and among all patients receiving modRNA therapy. Both controlled modRNA release into the cytoplasm following endocytosis and modRNA dose/protein effect relationships must be considered before modRNA can be implemented in cardiac therapy.

Although modRNA’s transient gene delivery eliminates the risk of malignancies associated with overexpression of genes delivered for long periods, further research is needed to improve modRNA’s translation efficiency in order to compensate for its short expression pattern and mitigate the need for repeated transfections. Further, plans to develop modRNA as a therapeutic intervention must consider the cost of production. Given the large amounts of modRNA needed to transfect organs as large the human heart (between 3 and 30 mg), [36] production costs must be reduced.

Regarding modRNA generation, the primary hurdle for mRNA therapy—its instability—has been effectively addressed. We hope that in the coming years, as research into modRNA targeting and delivery accumulates, reduced production costs and improved expression kinetics will accelerate the use of modRNA in numerous areas of medicine, including protein replacement therapies and genetic disorders.

**List of abbreviations**
Abbreviation: Full name
AC: Acid ceramidase
AAV: Adenovirus-associated virus
AV: Adenoviruses
ARCA: Anti-reverse cap analog
Ces1D: Carboxylesterase 1D
CM: Cardiomyocyte
CPP: Cell-penetrating peptides
EC: Endothelial cell
EAT: Epicardial adipose tissue
FLNP: Formulated lipidoid nanoparticles
HF: Heart failure
hFSTL1: Human follistatin-like
IGF1: Insulin-like growth factor-1
IHD: Ischemic heart disease
M3RNA: Microencapsulated modified mRNA
ModRNA: Modified mRNA
Pkm2: Pyruvate kinase muscle isoenzyme 2
TLRs: Toll-like receptors
aYAP: Yes-associated protein
